# The Impact of Physical Activity on Non-Motor Symptoms in Parkinson’s Disease: A Systematic Review

**DOI:** 10.3389/fmed.2016.00035

**Published:** 2016-08-17

**Authors:** Melanie E. Cusso, Kenneth J. Donald, Tien K. Khoo

**Affiliations:** ^1^School of Medicine, Griffith University, Gold Coast, QLD, Australia; ^2^Menzies Health Institute Queensland, Griffith University, Gold Coast, QLD, Australia

**Keywords:** Parkinson’s disease, non-motor symptoms, physical activity, systematic review, exercise

## Abstract

Parkinson’s disease (PD) is a neurological disorder that is associated with both motor and non-motor symptoms (NMS). The management of PD is primarily *via* pharmaceutical treatment; however, non-pharmaceutical interventions have become increasingly recognized in the management of motor and NMS. In this review, the efficacy of physical activity, including physiotherapy and occupational therapy, as an intervention in NMS will be assessed. The papers were extracted between the 20th and 22nd of June 2016 from PubMed, Web of Science, Medline, Ovid, SportsDiscuss, and Scopus using the MeSH search terms “Parkinson’s,” “Parkinson,” and “Parkinsonism” in conjunction with “exercise,” “physical activity,” “physiotherapy,” “occupational therapy,” “physical therapy,” “rehabilitation,” “dance,” and “martial arts.” Twenty studies matched inclusion criteria of having 10 or more participants with diagnosed idiopathic PD participating in the intervention as well as having to evaluate the effects of physical activity on NMS in PD as controlled, randomized intervention studies. The outcomes of interest were NMS, including depression, cognition, fatigue, apathy, anxiety, and sleep. Risk of bias in the studies was evaluated using the Cochrane Collaboration’s tool for assessing risk of bias. Comparability of the various intervention methods, however, was challenging due to demographic variability and methodological differences. Nevertheless, physical activity can positively impact the global NMS burden including depression, apathy, fatigue, day time sleepiness, sleep, and cognition, thus supporting its therapeutic potential in neurodegenerative conditions such as PD. It is recommended that further adequately powered studies are conducted to assess the therapeutic role of physical activity on both motor and non-motor aspects of PD. These studies should be optimally designed to assess non-motor elements of disease using instruments validated in PD.

## Introduction

Parkinson’s disease (PD) is a progressive bradykinetic disorder commonly presenting unilaterally, affecting over 1% of people over 55 years of age and marked by the degeneration of dopaminergic neurons in the substantia nigra (1, 2). While PD is most commonly associated with motor symptoms, such as tremor, rigidity, and gait disorders, there are numerous non-motor symptoms (NMS) associated with the condition such as hyposmia, constipation, cognitive impairment, anxiety, and depression (3). The treatment of the diverse array of NMS associated with PD can be challenging and non-pharmacological options such as education, support services, and exercise are likely to be underutilized due to various reasons that include limited knowledge on its therapeutic potential. In recent times, there is a growing body of evidence which supports the beneficial effects of non-pharmacological therapy, in particular, the effect of exercise on both motor and NMS (4).

Physical activity has been found to influence the brain’s neurochemistry and plasticity, through the upregulation of neurotrophins such as brain-derived nerve factor (BDRF) and nerve growth factor (NGF) in rat models (5, 6). BDRF has been suggested to increase turnover rate of dopamine *in vitro* and provide a neuroprotective role in nigral dopamine neurons (7). Additionally, BDRF has been thought to regulate branching and remodeling of axons and dendrites, whose length is reduced in PD (8, 9). NGF has also been linked to neuroprotection through stabilizing intracellular calcium which may influence L-type calcium channels know to mediate synaptic transmission of dopamine in rat midbrains (10, 11). The increased presence of such chemicals in the brain could help impede the progression of PD and potentially provide a neuroprotective effect.

Additionally, the relationship between exercise and endorphins has long been studied, as the opioid peptide can produce feelings of euphoria and well-being. The hormone is produced by the pituitary gland and hypothalamus during exercise, as well as excitement and pain (12). Endorphins are hypothesized to improve mood, such as depression and anxiety, *via* two different mechanisms; through binding to opioid receptors in the frontal cortex and limbic region, areas involved with mood; and by interacting with other neurotransmitters, such as dopamine, which also partake in improvement of mood (13).

Physical activity is becoming more popular for the treatment of chronic disease such as PD. However, the focus of many studies, to date, has primarily been on motor symptoms, with fewer studies assessing its effect on NMS. This review is aimed at determining the best available current evidence on the effects of various forms of exercise on NMS in PD.

## Methods

### Literature Search

This review included articles which met all of the following criteria: 10 or more participants diagnosed with idiopathic PD completing the intervention, participants needed to be randomized, and studies must evaluate the effect of physical activity on NMS in PD. For the purpose of this review, physical activity is defined as “any bodily movement produced by skeletal muscles that result in energy expenditure” (14). Papers were excluded if published prior to 1996 and did not assess global or specific NMS as a variable of interest. Additionally, papers needed to score a minimum score of 2 out of a maximum 5 according to the JADAD Scale, a short and widely used method to assess the quality of a report (15). The literature search was conducted using six databases between the 20th and 22nd of June 2016 (PubMed, Medline, Ovid, SportsDiscuss, Scopus, and Web of Science). Search terms included the MeSH terms for “Parkinson’s,” “Parkinson,” and “Parkinsonism” combined with “exercise,” “physical activity,” “dance,” “physiotherapy,” “occupational therapy,” “physical therapy,” “rehabilitation,” and “martial arts.” Titles and abstracts were read with papers not meeting selection criteria being discarded, and those remaining were read in full to check for suitability, in accordance with the Preferred Reporting Items for Systematic and Meta-Analyses (PRISMA) (see Figure S1A in Supplementary Material) (16). The protocol for this systematic review was registered with PROSPERO on the 8th of April 2016.

### Data Extraction

Data extraction was completed by one reviewer confirmed by a fellow author. Relevant articles meeting the inclusion criteria were reviewed with all relevant information, such as type of intervention, frequency, duration, and mode of delivery, along with outcomes.

### Outcomes of Interest

The outcomes of interest were both global and specific NMS. Those assessed globally included instruments such as the Unified Parkinson’s Disease Rating Scale Part 1 (UPDRS-1) and the Non-Motor Symptom Scale (NMSS), as well as more focused assessments of cognition, depression, fatigue, apathy, anxiety, and sleep using validated instruments.

## Results

The database search yielded 20 papers which met inclusion criteria (see Table 1).

**Table 1:** **Study characteristics**.

Reference	Participants	Dropouts (*N*)	Frequency	Length of session	Duration of intervention	Activity type	Design	JADAD score
Burini et al. (35)	29	AeT = 2	3/week	45 min	7 weeks	– Aerobic training (AeT) – Qigong (GQ)	Randomized (PD) cross over trial	5
		GQ = 2						
Clarke et al. (26)	39	OT = 1	3/month	45 min	2 months	– Occupational therapy (OT)	Randomized control (PD) – no exercise	5
		Control = 1						
David et al. (29)	51	PRET = 5	2/week	Not specified	24 months	– Progressive resistance exercise training (PRET) – Modified fitness counts (mFC)	Randomized (PD) into 2 intervention groups	5
		mFC = 7						
Duncan and Earhart (21)	62	AT = 16	2/week	1 h	12 months	– Argentine tango (AT)	Randomized control (PD) – no exercise	5
		Control = 11						
King et al. (34)	78	Home = 0	3/week	1 h	4 weeks	– Home exercise program – Individual exercise program – Group exercise program	Randomized (PD) into 3 intervention groups stratified by comorbidity level	5
		Individual = 0						
		Group = 1						
Modugno et al. (32)	24	Physio = 2	– Physio: 3/week – AT: 2–4/month	– Physio: 2–3 h – AT: 6 h	3 years	– Physiotherapy – Active theater training	Randomized controls (PD) – physiotherapy, stratified by age, sex, years of PD diagnosis and pharmacological treatment	5
		Active T = 2						
Nadeau et al. (19)	45	STT = 16	3/week	1 h	24 weeks	– Speed treadmill training (STT) – Mixed treadmill training (MTT)	Randomized control (PD) – no exercise	5
		MTT = 19						
		Control = 22						
Pohl et al. (17)	18	RGR = 0	2/week	1 h	6 weeks	– Ronnie Gardiner Rhythm and Music Method (RGR)	Randomized control (PD) – no exercise	5
		Control = 2						
Shulman et al. (28)	80	HIT = 3	3/week	Varied	3 months	– High-intensity treadmill training (HIT) – Low-intensity treadmill training (LIT) – Stretching and resistance training (S–R)	Randomized (PD) into 3 intervention groups	5
		LIT = 4						
		S–R = 5						
Sturkenboom et al. (18)	191	OT = 3	Varied	Mostly 1 h	10 weeks	– Occupational therapy (OT)	Randomized control (PD) – no exercise using minimization algorithm	5
		Control = 6						
Winward et al. (31)	39	Gym = 0	Varied	30–45 min	12 weeks	– Gym-based exercise program	Randomized with non-gym control	5
		Control = 0						
Bridgewater and Sharpe (30)	26	Aerobic = 0	2/week	1 h	12 weeks	– Aerobic exercise	Randomized with inactive control	4
		Control = 0						
Nocera et al. (27)	23	TC = 2	3/week	1 h	16 weeks	– Tai Chi (TC)	Randomized control (PD) – non-contact	4
		Control = 0						
Teixeira-Machado et al. (36)	30	FPTP = 0	2/week	1 h	25 weeks	– Feldenkrais physical therapy program (FPTP)	Randomized control (PD) – educational lectures	4
		Control = 0						
Xiao and Zhuang (24)	96	BQ = 3	– BQG: 4/week – Walking: daily	– BQG: 15 min – Walking: 30 min	6 months	– Baduanjin Qigong with walking (BQ) – Walking	Randomized control (PD) – walking	4
		Walking = 4						
Cholewa et al. (20)	70	Physio = 0	2/week	1 h	12 weeks	– Physiotherapy	Randomized control (PD) – no exercise	2
		Control = 0						
Cugusi et al. (25)	20	NW = 0	2/week	1 h	12 weeks	– Nordic walking (NW)	Randomized control (PD) – no exercise	2
		Control = 0						
Miyai et al. (33)	24	Treadmill = 1	3/week	45 min	1 month	– Body weight-supported treadmill training	Randomized with conventional physical therapy control	2
		Physio = 3						
Park et al. (23)	31	ESG = 1	3/week	1 h	48 weeks	– Early start exercise (ESG) – Delayed start exercise (DSG)	Randomized (PD) into 2 intervention groups	2
		DSG = 0						
Rios Romenets et al. (22)	33	Tango = 0	2/week	1 h	12 weeks	– Argentine tango (AT)	Randomized control (PD) – self-directed exercise	2
		Control = 1						

### Participants

The number of participants in the studies ranged from 18 (17) to 191 (18). The majority of studies had a higher male to female ratio (18–31), apart from two which had equal proportions (32, 33) and three which had a higher proportion of females (17, 34, 35). One study did not mention the ratio of male to female participants (36). The overall age range was from 40 to 89 years (17–36).

### Intervention and Activity Type

The majority of studies conducted 2–4 sessions/week, each lasting between 20 and 90 min (17, 19–23, 25–31, 33–36), with some studies opting for increased or decreased frequency and length (18, 24, 32). The total intervention period varied greatly, with the shortest duration being 4 weeks (34), and the longest being 3 years (32). The most common duration was 12 weeks, with five studies opting for that duration (20, 22, 25, 30, 31). Eleven studies had a short intervention period between 4 and 12 weeks (17, 18, 20, 22, 25, 26, 30, 31, 33–35), six had medium intervention durations between 3 and 6 months (19, 23, 24, 27, 28, 36), and three had long intervention periods of 1 year (21), 2 years (29), and 3 years (32). The studies all utilized an active intervention, the physical activity including aerobic training (30, 35), treadmill training (19, 28, 33), and walking (24); resistance training (28, 29); balance training, Tai Chi (20, 27), and Baduanjin Qigong (24, 35); as well as customized programs such as physiotherapy (32), OT (18, 26), physiotherapist-supervised exercise, self-supervised exercise (34), group exercise (34), active theater training (32), Argentine tango (21, 22), early and delayed start exercise modified for PD (23), Feldenkrais physical therapy program (36), Nordic Walking (25), modified fitness counts (29), gym-based exercise program (31), and the Ronnie Gardiner Rhythm and Music Method (17) (see Table 2).

**Table 2:** **Non-motor outcomes of studies**.

Reference	Outcome	Change	*P*-value
Cholewa et al. (20)	– UPDRS I	Physio = −0.45 points, control = 0.1 points	**0.001**
Cugusi et al. (25)	– NMSS	NW = −23.2 points, control = 1 point	<0.05
	– Fatigue (PFS-16)	NW = −11.7 points, control = 0.6 point	**<0.05**
	– Depression (BDI-II)	NW = −5.2 points, control = 0.4 point	**<0.005**
	– Apathy (SAS)	NW = −6.3 points, control = 1 point	**<0.0005**
Duncan and Earhart (21)	– MDS-UPDRS I	Tango = −1.62 points, control = 0.42	NS
Modugno et al. (32)	– UPDRS-1	Theater = −0.8 points, control = 0.3 points	**<0.05**
	– Depression (HDRS)	Theater = −12.3 points, control = 0.9 points	**<0.001**
	– Daytime sleepiness (ESS)	Theater = −7 points, control = −0.3 points	**<0.001**
Miyai et al. (33)	– UPDRS-1	BWSTT = −0.5 points, control = −0.2 points	NS
Nadeau et al. (19)	– MDS-UPDRS I	Control = −0.7 points, speed TT = −0.4 points, mixed TT = −0.5 points	0.93
	– Depression (BDI-II)	Control = −0.6 points, speed TT = −5.8 points, mixed TT = −1.2 points	0.09
	– Cognition (MMSE)	Control = −0.1 points, speed TT = 0.7 points, mixed TT = −0.2 points	0.12
Bridgewater and Sharpe (30)	– Depression (LPDQ)	Exercise = non-depressed both pre and post intervention, control = non-depressed both pre and post intervention	NS
Burini et al. (35)	– Depression (BDI)	Group AT1-GQ2: AT = 1 point, GQ = −1 point	NS
		Group GQ1-AT2: AT = 3 points, GQ = −4 points	
Clarke et al. (26)	– Anxiety (HADS)	OT = 1.44 points	N/A^#^
	– Depression (HADS)	OT = −1.42 points	N/A^#^
David et al. (29)	– Cognition (digit span forwards and backwards)	mFC = 0.7 points, PRET = 0.5 points	0.27
	– Cognition (Stroop test)	mFC = 0.3 points, PRET = 0.2 points	0.77
	– Cognition (BTA)	mFC = 0.1 points, PRET = 0.3 points	0.83
King et al. (34)	– Apathy (LARS)	Home = −0.41 points, individual = −2.24 points, group = −0.25 points	0.377
Nocera et al. (27)	– Cognition (digit span backward subtest)	TC = 0.5 points, control = −0.7 points	0.08
	– Cognition (letter verbal fluency)	TC = 2.4 points, control = −1.3 points	0.39
	– Cognition (categorical verbal fluency)	TC = 1.9 points, control = −0.5 points	0.64
	– Cognition (Stroop test score)	TC = 3.8 points, control = 0.8 points	0.75
	– Cognition (Trail Marking A)	TC = −11.5 s, control = −0.2 s	0.24
	– Cognition (Trail Marking B)	TC = −15.4 s, control = −7.8 s	0.52
Park et al. (23)	– Depression (BDI)	ESG = −2.67 points, DSG = −1.6 points	**0.04**
Pohl et al. (17)	– Cognition (text recall test)	RGR = 3.5 points, control = 2.3 points	0.63
	– Cognition (symbol digit modification)	RGR = 1.5 points, control = 3.5 points	0.18
	– Cognition (Clox and Cube)	RGR = 0 points, control = −0.5 points	0.21
	– Cognition (Naming 30 items)	RGR = 0.5 points, control = 1 point	1
	– Cognition (Stroop test time)	RGR = −2 s, control = −0.5 s	0.54
	– Cognition (PaSMO)	RGR = −6.5 s, control = −22 s	0.13
Rios Romenets et al. (22)	– Cognition (MoCA)	Total: tango = 0.4 points, control = −0.6 points	0.080
		Visuospatial/executive function: tango = 0.1 points, control = −0.2 points	0.362
		Attention: tango = 0.2 points, control = 0 points	0.419
		Delayed recall: tango = 0.3 points, control = −0.2 points	0.223
	– Depression (BDI)	Tango = −0.2 points, control = −0.4 points	0.770
	– Fatigue (KFSS)	Tango = −3.5 points, control = 2.6 points	0.057
	– Apathy (AS)	Tango = 2.4 points, control = 2.6 points	0.904
Shulman et al. (28)	– Depression (BDI)	HIT = 1.43 points, LIT = −0.68 points, S–R = 0.68 points	NS^#^
	– Fatigue (PFS-16)	HIT = 0.52 points, LIT = 1.73 points, S–R = 0.55 points	NS^#^
Sturkenboom et al. (18)	– Fatigue (FSS)	OT = 0.1 points, control = 0 points	0.846
	– Mood (BDI)	OT = −1 point, control = −1 point	0.318
Teixeira-Machado et al. (36)	– Depression (BDI)	Depression scores improved for the Feldenkrais group and decreased for the control group	**0.05**
	– Cognition (MMSE)	FG = 2.1 points, control = −1.18 points	**0.0007**
Winward et al. (31)	– Fatigue (FSS)	Gym exercise program = −0.4 points, control = −0.36 points	NS
Xiao and Zhuang (24)	– Sleep (PDSS-2)	Total: BQ = −13.72 points, walking = −2.04 points	**0.045**
		Motor symptoms at night: BQ = −5.59 points, walking = −0.75 points	**0.049**
		PD symptoms at night: BQ = −3.28 points, walking = −0.26 points	**0.037**
		Disturbed sleep: BQ = −3.76 points, walking = −0.35 points	**0.045**
	– Fatigue (PFS-16)	BQ = −0.31 points, control = −1.08 points	0.526

*Instrument abbreviations: UPDRS, Unified Parkinson’s Disease Rating Scale; BDI, Beck Depression Inventory; MMSE, Mini Mental State Examination; HDRS, Hamilton Depression Rating Scale; ESS, Epworth Sleepiness Scale; NMSS, Non-Motor Symptom Scale; PFS-16, Parkinson’s Fatigue Scale-16 Questions; SAS, Starkstein Apathy Scale; PDSS-2, Parkinson’s Disease Sleep Scale Version 2; MoCA, Montreal Cognitive Assessment; KFSS, Krupp’s Fatigue Severity Scale; AS, Apathy Scale; LARS, Lille Apathy Rating Scale; PaSMO, Parallele serial mental operations; FSS, Fatigue Severity Scale; HADS, Hospital Anxiety and Depression Scale; BTA, Brief Test of Attention*.

*Significant values are in bold*.

*^#^Marks intra group P-value; NS, non-significant; N/A, not available*.

*Negative score indicates improvement: UPDRS-1, BDI, HDRS, ESS, PFS-16, SAS, PDSS-2, KFSS, AS, LARS, PaSMO, FSS HADS, Stroop test time, and trail making*.

*Positive score indicates improvement: MMSE, NMSS, MoCA, BTA, digit span, Stroop test score, letter verbal fluency, categorical verbal fluency, text recall, symbol digit modification, Clox and Cube, and naming 30 items*.

### Medication

Nine of the studies analyzed the participants on medication (17, 22, 25, 27, 28, 31, 33–35); however, three of those had a change in medication as exclusion criteria (17, 22, 33). Three studies (21, 24, 29) assessed the participants off their medication, one (23) did not include anyone on levodopa, and seven studies did not specify participant medication status (18–20, 26, 30, 32, 36).

### Failed to Complete Study

Five of the studies did not have failures to complete (20, 25, 30, 31, 36), while 15 did have participants not completing the study (17–19, 21–24, 26–29, 32–35). Reasons for failure to complete were not wanting to continue (18, 21), scheduling issues (18, 19, 21, 27), commute difficulties (28), changes in medication (17, 19, 33), other comorbidities (17–19, 21, 28), received other intervention simultaneously (18), musculoskeletal injuries (19, 35), motor vehicle accident (19), unreturned questionnaires (26), could not continue due to health reasons (22), poor compliance (23, 35), inability to commit to sessions (34), hospitalization (24), too much to handle, physical decline, physically unable, underwent deep brain stimulation, moved away (21), family demands (21, 28, 34), and no clear explanation (18, 22, 24). One study did not outline reasons for dropouts (29).

### Measurement Tools

Global NMS were measured with UPDRS part 1 or the revised MDS-UPDRS part 1 (19–21, 32, 33), and the NMSS (25). Depression was measured using the Beck Depression Inventory (BDI/BDI-II) (18, 19, 22, 23, 25, 28, 35, 36), the Hamilton Depression Rating Scale (Ham-D) (32), the Levine–Pilowsky Depression Questionnaire (LPDQ) (30), and the Hospital Anxiety and Depression Scale (HADS) (26). Cognition was evaluated using the Mini Mental State Exam (MMSE) (19, 36), the Montreal Cognitive Assessment (MoCA) (22), subsections of the Cognitive Assessment Battery (CAB) (17), Stroop Test (17, 27, 29), and the Brief Test of Attention (BTA) (29). Some studies chose to use subsections of the cognitive tests as previously mentioned (27, 29). Daytime sleepiness was measured with Epworth Sleepiness Scale (ESS) (32), and sleep quality with Parkinson Sleep Scale (PSS) (24). Fatigue was analyzed by the 16-item Parkinson’s Disease Fatigue Scale (PFS-16) (24, 25, 28), Fatigue Severity Scale (FSS) (18, 31), and the Krupp Fatigue Severity Scale (KFSS) (22), whereas apathy was measured using the Starkstein Apathy Scale (SAS) (25), the Apathy Scale (AS) (22), and the Lille Apathy Rating Scale (LARS) (34). For further detail, see Table 2.

### Methodology Quality

The studies selected were randomized (17–36), one study used a crossover design (35), four studies randomized into multiple intervention groups (23, 28, 29, 34), while the rest used as a control group (17, 18, 20–22, 24–27, 30–33, 36). Three of the studies stratified the groups for intervention (31, 32, 34). Sixteen of the studies had a blinded component, with 1 (19) being double blinded as participants were unaware which group they were randomized to (speed or mixed treadmill training) and 15 being assessor blind (17, 18, 21, 23, 24, 26–32, 34–36). The lack of an inactive PD control in some studies may have contributed to bias, along with the studies which were unblinded.

### Risk of Bias

Risk of bias was assessed using the Cochrane tool for assessing risk of bias, which assesses bias in a number of different aspects that include random sequence generation, allocation concealment, blinding of participants and personnel, blinding of outcome assessment, blinding of outcome data, selective reporting, and other bias (37). Only four studies had a low risk of bias in all sections (19, 32, 34, 35), while majority had at least one criterion with unknown bias (17, 18, 20, 23–29, 31, 33). Four studies did have high risk of bias in one section, three for selective reporting due to presenting only some of the data (21, 30, 36), and one for blinding of outcome assessment as assessors were not blinded to allocation (22). The criterion which was least reported, and thus had the highest number of unknown risk of bias was allocation concealment (17, 18, 20–31, 33, 36). For more information, see Figure S2A in Supplementary Material.

### Effect on Primary Outcomes

Significant improvements were found in three studies that assessed global NMS using UPDRS part 1, MDS-UDPRS part 1, or NMSS (20, 25, 32), with all other studies showing non-significant improvements (19, 21, 33). Depression also improved in 9 of the 10 studies (18, 19, 22, 23, 25, 26, 28, 32, 36), with statistically significant improvements in 4 studies (23, 25, 32, 36). Daytime sleepiness showed a significant improvement for active theater (32), and Baduanjin Qigong (24) significantly improved for both the overall score of the PDSS-2 and numerous subsections. Fatigue and apathy were significantly improved in one study (25).

## Discussion

In more recent times, non-pharmacological therapies in PD have become increasingly acknowledged as beneficial with various modalities offered to patient populations.

### Intervention Design

The study design varied greatly, in particular, when looking at frequency and length of intervention. Various studies showed a significant improvement in NMS, including two short duration (20, 25) and one medium duration studies (36) involving 2–3 sessions/week lasting 45 min to 1 h and a study with a low frequency of 6-h long classes (32). Depression was one of the most widely studied outcomes, showing significant improvements in a number of different activities (23, 25, 32, 36). Significant improvements were also seen in sleep (24, 32), fatigue, apathy (25), and cognition (36).

Although it is widely regarded that exercise affects brain plasticity, different exercise types have been found to selectively affect various brain regions. Aerobic training demonstrated its importance in the aging brain, showing the most benefit in brain regions most affected by aging, including prefrontal, superior parietal, and temporal cortices in gray matter, and anterior and transverse tracts between the frontal and parietal lobes in white matter, which are areas involved in cognition and everyday functioning (38). Another study on resistance training had shown to significantly change brain regions involved with response inhibition, including the left anterior insula which extends into the lateral orbital frontal cortex as well as the anterior part of the left middle temporal gyrus (39). Participants who underwent training twice weekly significantly improved in the Flanker test when compared with participants who had 12 months of twice weekly balance and tone training. However, participants who only performed resistance training once per week did not show significant improvements from the control group. This suggests that while different exercise types may beneficially affect different brain regions, the improvement was not distinguishable in this review as different types of exercise showed positive effects in global and specific NMS such as sleep. However, fatigue and apathy only showed significant improvement in aerobic exercise, possibly due to the brain regions primarily involved.

### Risk of Bias

Three of the studies showed a high risk of bias in selective reporting of outcome measures, highlighting the trends of importance to the authors, in particular, those with positive results (21, 30, 36). With regard to health-care interventions, it is important that all data are fully and clearly reported to help guide clinicians in decision-making (40). However, the majority of data were present or able to be retrieved from the authors in regard to the outcomes of interest of this study, alleviating the potential bias as a result of underreporting. One study did not blind the assessors for data collection, which may have implications on data collected (22). Unblinded assessors may exhibit prejudice to expectant results (41).

### Measurement Tools

Global measures of NMS were measured by UPDRS part1 (20, 32, 33), the MDS-UPDRS part 1 (19, 21), and NMSS (25). The UDPRS and the revised MDS-UPDRS are validated tools developed to have a compounded scale for numerous characteristics of PD (42–44). Additionally, one study used the NMSS, a validated comprehensive assessment of NMS in PD (45, 46).

In the MDS taskforce review of ASs, AS was ranked over LARS as its sensitivity was yet to be determined (47). The fatigue instruments analyzed by the MDS taskforce were the FSS and PFS, where FSS is validated for both screening and rating severity, while PFS was validated as screening tool. The KFS has also been independently validated for the screening of fatigue in chronic disease though not specifically validated in PD (48). Sleep instruments were also reviewed (49) with the ESS been shown to be both valid and reliable (50) and PDSS-2 also validated (51).

Validated depression scales reviewed and validated by the MDS taskforce included the BDI, HADS, and Ham-D. The Ham-D was shown to be an optimal instrument, followed by the BDI and HADS (52). HADS has also been suggested by the MDS taskforce for rating anxiety (53). The LPDQ has been validated for use in a general population and not in PD (54).

The MDS diagnostic criteria for mild cognitive impairment recommend that detailed level 2 assessment requires a minimum of two tests per cognitive domain, but stated that excessive or imbalanced number of tests per cognitive domain could cause bias (55). The MoCA has adequate metrics as a brief assessment of global cognition in PD (56), whereas the MMSE is thought to be less sensitive to mild cognitive impairment in PD (57). The CAB has been shown to be sensitive in mild cognitive impairment and dementia but is yet to be validated in PD (58).

### Other Conditions

Numerous other conditions, including the intervention environment, group, or individual exercise, if a family or friend was present, patient’s location and the person leading the interventions were considered to see if other circumstances might have influenced the results. Thirteen of the papers mentioned the environment (18, 19, 21, 22, 24, 25, 28, 30, 31, 33, 34, 36), 16 commented on the supervisor/facilitator of the intervention (17, 18, 20, 22–34), 2 specified if a companion was present (18, 22), and 1 gave a clear living location of the participants (18).

The environment can have numerous effects on NMS such as cognition. One study found that interaction with natural environments as opposed to urban environments can improve cognitive control (59). This could provide a basis for why Nordic walking, which was performed at a city park, had significant improvements in the NMS tested (25). Group settings and family support can also contribute to aspects such as exercise frequency and involvement. It was previously found that social support networks are low in older adults compared with younger individuals, possibly due to issues such as peers becoming less active and family falling into social stereotypes fearing injury for a frail elderly population (60). This could suggest that studies which comprise group settings, as well as including family members, could be beneficial to the participant.

### Limitations

This review does present a number of limitations. First, the study sample size was modest, with 20 papers reviewed, in-part due to the strict criterion. Additionally, the majority of papers include aerobic or custom training programs, with only a small fraction looking at strength and balance training. Furthermore, variable assessment of NMS using different instruments made it challenging for comparing study outcomes. Due to these limitations, a meta-analytical approach to this topic will be suitable when more well-designed studies are made available.

## Conclusion

Physical activity may be a suitable non-pharmacological therapy in PD. Global and specific NMS involving depression, apathy, fatigue, cognition, and sleep were significantly improved by some form of physical activity. However, the synergistic effects of both pharmacological and non-pharmacological treatments in PD are still unclear. For an unbiased appraisal of each activity type, further research is needed which prioritizes NMS to help determine the most beneficial effects of physical activity in this complex condition.

## Author Contributions

MC conducted the literature review and drafted the manuscript. KD reviewed the manuscript. TK supervised and reviewed the manuscript.

## Conflict of Interest Statement

The authors declare that the research was conducted in the absence of any commercial or financial relationships that could be construed as a potential conflict of interest.
