# Supplementary figures and images for: The Impact of Physical Activity on Non-Motor Symptoms in Parkinson’s Disease: A Systematic Review
Source: Front Med (Lausanne). 2016 Aug 17;3:35. doi: 10.3389/fmed.2016.00035 (PMC4987718; doi:10.3389/fmed.2016.00035)

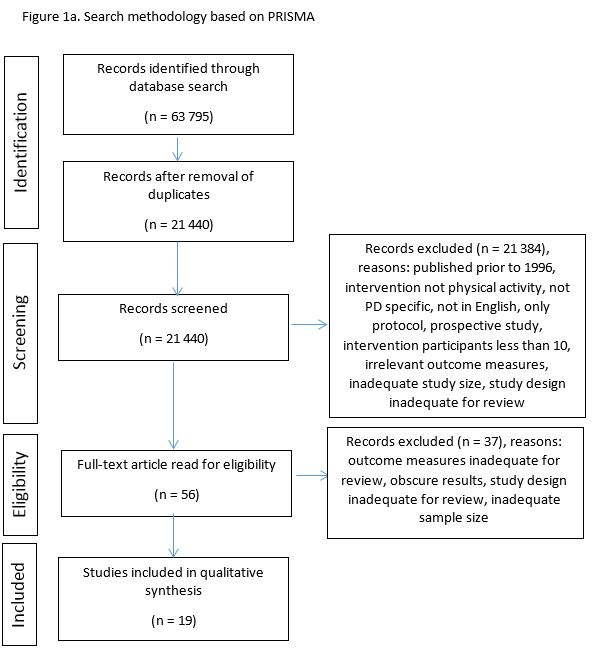

Supplement: Supplementary file 1 [file Figure_1.JPEG]

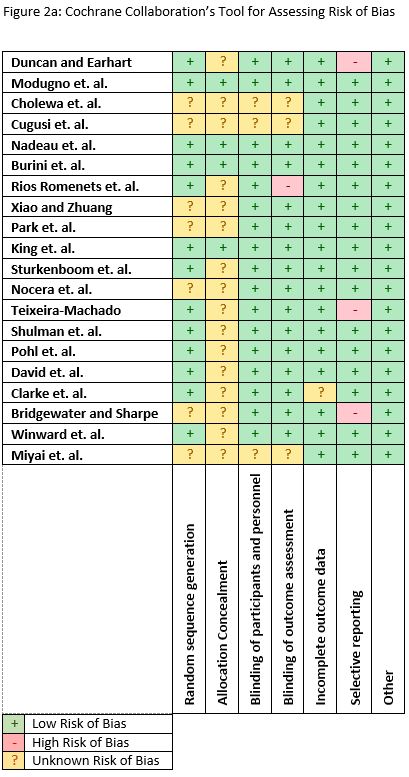

Supplement: Supplementary file 2 [file Figure_2.JPEG]
